# Surgical Outcomes in Radiation-induced Cataracts After External-beam Radiotherapy in Retinoblastoma

**DOI:** 10.4274/tjo.70019

**Published:** 2018-06-28

**Authors:** Şerife Bayraktar, Samuray Tuncer, Cahit Özgün, Gönül Peksayar, Rejin Kebudi

**Affiliations:** 1 İstanbul University İstanbul Faculty of Medicine, Department of Ophthalmology, İstanbul, Turkey; 2 İstanbul University Institute of Oncology, Department of Pediatric Oncology, İstanbul, Turkey

**Keywords:** Retinoblastoma, radiotherapy, cataract, phacoemulsification

## Abstract

**Objectives:**

To investigate visual outcomes, surgical complications and tumor recurrence among children with retinoblastoma undergoing phacoemulsification and posterior chamber intraocular lens (PCIOL) implantation for radiation-induced cataract secondary to external beam radiotherapy.

**Materials and Methods:**

The medical records of all patients treated by phacoemulsification and PCIOL implantation for radiation-induced cataract after external beam radiotherapy for retinoblastoma at a single institution between 1980 and 2014 were reviewed retrospectively. The study included 6 eyes of 6 children (4 girls, 2 boys).

**Results:**

Four patients had bilateral and two patients had unilateral retinoblastoma. The median age at diagnosis of retinoblastoma was 28.3 months (range, 12-96 months). All patients received chemoreduction (OPEC protocol) and external beam radiotherapy with or without local ophthalmic therapies and developed radiation-induced cataracts. The median interval from retinoblastoma diagnosis to cataract surgery was 96.3 months (range, 73-122 months). Time interval between surgery and last retinoblastoma treatment was 67.2 months. Postoperative complications included iridocyclitis in 2 eyes and posterior capsule opacification in all eyes. The mean follow-up after surgery was 105.8 months (range, 59-120 months). Final visual acuity was better in all eyes than preoperative visual acuities.

**Conclusion:**

Phacoemulsification and PCIOL implantation is an effective method of managing radiation-induced cataracts in eyes with previously treated retinoblastoma. However, visual acuity was limited by the presence of primary macular tumor.

## Introduction

Retinoblastoma is the most common malignant intraocular tumor in childhood, with a prevalence of approximately 1 in 20,000.^1^ Treatment methods for retinoblastoma include systemic chemotherapy, local chemotherapy, photocoagulation, cryotherapy, thermotherapy, brachytherapy, external radiotherapy, enucleation and exenteration.^2,3,4^ Retinoblastoma is radiosensitive tumor, requiring a dose of 35-45 Gy.^3,5^ However, 2 Gy is enough to cause cataracts in the crystalline lens. Therefore, cataract formation is a leading complication, along with retinopathy, orbital hypoplasia and secondary tumor development.^3,4,5^ Because cataract both impairs vision and prevents fundus examination, surgery is unavoidable.

Numerous studies have indicated that surgical removal of radiation cataracts in children with retinoblastoma does not generally cause tumor spread or new tumor formation. The aim of this study was to evaluate the visual outcomes, complications and tumor recurrence rates after phacoemulsification and posterior chamber intraocular lens (PCIOL) implantation in children with radiation-induced cataracts.

## Materials and Methods

The records of 206 patients who were diagnosed and treated for retinoblastoma in the Tumor Unit of the İstanbul University İstanbul Faculty of Medicine, Department of Ophthalmology between 1980 and 2014 were retrospectively reviewed. Of these, 6 eyes of 6 patients who received radiotherapy and later underwent phacoemulsification and PCIOL implantation due to radiation-induced cataract were separately evaluated. The study was approved by the local ethics committee and conducted in accordance with the principles set forth in the Declaration of Helsinki.

The patients’ records were screened for demographic data, age at retinoblastoma diagnosis, affected side, hereditary pattern, stage (Reese-Ellsworth and International Classification), macular involvement, treatments received, type and dose of radiotherapy received, time between radiotherapy and cataract development, date of surgery, time between last retinoblastoma treatment and cataract surgery, type of surgery, type of intraocular lens, intra- and postoperative complications, pre- and postoperative best corrected visual acuity (BCVA), postoperative tumor recurrence or spread, postoperative follow-up time and any other postoperative interventions.

All patients were operated due to significant visual impairment and difficulty examining the fundus. Surgeries were performed after a mean period of 67 months with no tumor progression.

Prior to surgery, all patients underwent keratometry and axial length was calculated with ultrasound biometry. For patients with macular involvement, axial length was measured from their fellow eyes. Phacoemulsification (Advanced Medical Optics Sovereign, Santa Ana, California, USA) and PCIOL implantation through a clear corneal incision were performed under general anesthesia. The posterior capsule was left intact in all cases because the patients were at least 90 months old at the time of surgery and Nd:YAG laser could be applied afterwards. This prevented the possibility of retinal detachment, because both tumor spread to the anterior chamber and anterior vitrectomy could result in traction in the retina.

## Results

Two hundred seventy-six eyes of 206 patients who were diagnosed and treated for retinoblastoma were retrospectively evaluated. Considering the complications of radiotherapy, external-beam radiotherapy was only applied to 40 eyes of 35 patients; of these, 13 eyes developed cataract. Six eyes with cataract that severely reduced visual acuity and prevented fundus examination underwent phacoemulsification and PCIOL implantation.

Four of the patients were female and 2 were male. Two patients had unilateral retinoblastoma and 4 patients had bilateral retinoblastoma. Only one patient had familial retinoblastoma, while the others were sporadic. The average age at retinoblastoma diagnosis was 28.3 months (12-96 months). According to the Reese-Ellsworth classification, tumor stage was 5B in 1 eye, 3B in 4 eyes and 1B in 1 eye.

All patients received systemic OPEC protocol (vincristine 1.5 mg/m^2^ [O], cisplatin 80 mg/m^2^ [P], etoposide 200 mg/m^2^ [E], cyclophosphamide 600 mg/m^2^ [C]) chemotherapy. A radiotherapy dose of 35-40 Gy was delivered over 18-22 sessions. The mean time from last radiotherapy treatment to cataract development was 31.6 months. The mean age at surgery was 124.6 months (90-169). The mean time from last retinoblastoma treatment and surgery was 67.2 months.

Six eyes of 6 patients underwent phacoemulsification and PCIOL implantation via a clear corneal incision under general anesthesia. Foldable acrylic intraocular lenses were implanted. The posterior capsule was left intact in all eyes. None of the patients developed intraoperative complications. Two eyes developed iridocyclitis postoperatively but it responded to topical treatment. When fundus examination became possible postoperatively, radiation retinopathy was detected in one patient.

Posterior capsule opacification was observed in all eyes at a mean of 10.8 months postoperatively and Nd:YAG laser capsulotomy was performed on 4 eyes at a mean of 70.7 months postoperatively. None of the patients had elevated intraocular pressure or retinal detachment.

Five patients exhibited macular involvement and their preoperative BCVA was counting fingers from an average of 1.6 m. One eye with extramacular involvement had a preoperative BCVA of 0.16. Although visual acuity increased in all eyes postoperatively, improvement was limited due to the macular involvement. The preoperative and postoperative BCVAs and other characteristics of the patients are summarized in Table 1.

The mean postoperative follow-up period was 105.8 months (59-120 months). There was no tumor recurrence or progression in any of the patients during follow-up.

## Discussion

Because retinoblastoma is a radiosensitive tumor, external beam radiotherapy was among the first-line treatment options for retinoblastoma for much of the 20^th^ century.^3,4,5^ However, due to radiation-induced complications such as cataract, retinopathy, orbital hypoplasia and secondary tumor development, chemotherapy began to take the place of radiotherapy starting in the early 1990s and radiotherapy became a second-line option for chemoresistant tumors with multiple foci and diffuse vitreous and/or subretinal seeding.^3,5,6^ Although the lens-protective radiotherapy technique has reduced radiation-induced cataract, eyes treated with radiotherapy still develop cataracts at rates between 22% and 78%.^7,8,9^ In our study, of the 276 eyes of 206 patients diagnosed with retinoblastoma in our clinic between 1980 and 2014, only 40 underwent radiotherapy and 13 (32%) of those eyes developed cataracts.

Reese^10^ first published surgical outcomes in radiation-induced cataracts in 1939. Reese^10^ performed intracapsular cataract extraction and reported mostly inflammation-related complications due to residual cortex fragments. In 1998, Portellos and Buckley^11^ evaluated 11 eyes of 8 patients who underwent extracapsular cataract extraction and PCIOL implantation and reported that inflammation and fibrin membrane formation occurred postoperatively in 3 eyes but regressed with treatment. Miller et al.^12^ reported in 2005 that postoperative iridocyclitis occurred at a rate of 19% following pars plana lensectomy (PPL), PCIOL implantation and pars plana vitrectomy in 16 eyes of 12 patients. Our patients underwent phacoemulsification and PCIOL implantation with intact posterior capsules. Iridocyclitis was observed postoperatively in 2 eyes but improved with topical treatment. Although there were few patients in our study, we can conclude that postoperative inflammation is less severe after procedures in which the posterior capsule is preserved and the iris plane is avoided, as well as those not using a pars plana approach.

One of the most important points in pediatric cataract surgery is not leaving the posterior capsule intact. Especially with IOL implantation, secondary cataracts are common and can lead to amblyopia due to visual axis obscuration.^13^ In eyes treated for retinoblastoma, however, the posterior capsule is believed to possibly serve as a barrier, preventing tumor spread to the anterior segment. For this reason, Hoehn et al.^14^ performed lens aspiration and PCIOL implantation and left the posterior capsule intact in their series of 19 patients. Twelve (63.2%) of the eyes developed posterior capsule opacification and underwent Nd:YAG laser capsulotomy. Payne et al.^15^ performed extracapsular cataract extraction through a limbal-based scleral tunnel on 12 eyes, choosing to do posterior capsulotomy and anterior vitrectomy in 7 of the eyes because of the dense subcapsular plaque in the posterior capsule, while leaving the posterior capsule intact in the other 5 eyes. They later performed Nd:YAG laser capsulotomy on these 5 eyes. We also left the posterior capsule intact in our patients, both because their age was over 90 months and considering the barrier function of the posterior capsule. Posterior capsule opacification developed in all of our patients, which we treated with Nd:YAG laser with no complications.

Although retinal detachment is among the complications that may develop due to vitreous traction in patients who undergo posterior capsulotomy and anterior vitrectomy, Brooks et al.^16^ reported retinal detachment in only 1 patient after PPL and anterior vitrectomy in 42 eyes of 38 patients. We also observed no postoperative retinal detachment in our patients.

One of the most important parameters after surgery for radiation cataract in retinoblastoma patients is the presence or absence of tumor recurrence. Brooks et al.^16^ reported 3 tumor recurrences in their series of 42 eyes. They attributed these recurrences to having performed PPL and to the presence of post-radiotherapy haze or vitreous hemorrhage in the vitreous during cataract surgery. In 2005, Hanovar et al.^17^ presented the surgical outcomes of 34 eyes of 34 patients. They performed intracapsular cataract extraction on 1 patient, extracapsular cataract extraction on 28 patients and PPL on 5 patients and observed tumor recurrence in 5 cases. They noted that the average time from last retinoblastoma treatment to surgery was 6 months for patients who developed recurrence and 26 months for the other patients. Moshfeghi et al.^18^ reported tumor recurrence in 1 of their 4 patients, who had to undergo enucleation. Osman et al.^19^ reported tumor recurrence in 3 of 21 patients who underwent lens aspiration, posterior capsulotomy and anterior vitrectomy through a clear corneal incision approach, with 2 requiring enucleation. They believed recurrence in these patients was related to the advanced disease stage and the fact that they performed posterior capsulotomy. They reported that the only difference in these patients was that the time between cataract surgery and last treatment was 12 months. Although the interval is not known clearly, Portellos and Buckley^11^ and Miller et al.^12^ reported no tumor recurrence in their patients and the time between last retinoblastoma treatment and surgery was at least 16 months.

Hoehn et al.^14^ proposed that the lack of tumor recurrence in their study was due to having left the posterior capsule intact. Payne et al.^15^ contend that surgery with a scleral tunnel approach is safer and that in this way, there will be no surgical site leakage due to eye scratching, especially in children. Particularly important is definitive tumor control and, according to the literature, the need to wait for at least 9 months prior to surgery. No tumor recurrences were observed in any of our cases despite the very long (mean 105.8 months) postoperative follow-up period, likely due to long interval between the last retinoblastoma treatment and surgery (mean 67.2 months) and the intact posterior capsule.

Even with uncomplicated cataract surgery, final visual acuity is dependent on whether the macula is involved. In a series of 21 cases reported by Osman et al.,^19^ postoperative visual acuity was 20/20 in only 4 patients, between 20/20 and 20/200 in 9 patients and lower than 20/200 in the remaining patients. Of the patients with low vision, 3 had macular involvement, 2 had radiation keratopathy, 2 were enucleated due to tumor recurrence and 1 developed neovascular glaucoma. Hoehn et al.^14^ reported low vision in 5 patients with macular involvement and in another 4 patients due to radiation keratopathy and retinopathy. Miller et al.^12^ reported that cystoid macular edema and postoperative inflammation caused decreased vision but the patients did not completely lose their vision. Portellos and Buckley^11^ did not observe low vision in any of their patients but their follow-up was shorter compared to the other studies (mean 20 months). Brooks et al.^16^ reported patients with radiation keratopathy and radiation retinopathy but did not explain whether this led to decreased vision. Shanmugen et al.^20^ reported vision loss due to radiation maculopathy 3 years after surgery. In our case series, we observed that 2 patients with preoperative BCVA of CF 1 m had a postoperative BCVA of CF 2 and 3 m, while BCVA increased from CF 0.6 m to CF 2 m, from CF 1.5 m to 0.05 and from CF 4 m to 0.125 in 3 other patients. Visual acuity was limited in these 5 patients due to macular involvement. Another patient with no macular involvement and a preoperative BCVA of 0.16 had a postoperative BCVA of 0.6. Ultimately, visual acuity increased in all of our patients.

## Conclusion

In conclusion, phacoemulsification and PCIOL implantation with clear corneal incision approach and leaving the posterior capsule intact is a reliable method for radiation cataract surgery in retinoblastoma patients. Although this retrospective study did not include a large number of patients, we can conclude that surgical intervention done after ensuring retinoblastoma is controlled with treatment and delayed at least 9 months is safe in terms of tumor recurrence. However, macular involvement limits improvement of visual acuity.

## Figures and Tables

**Table 1:**
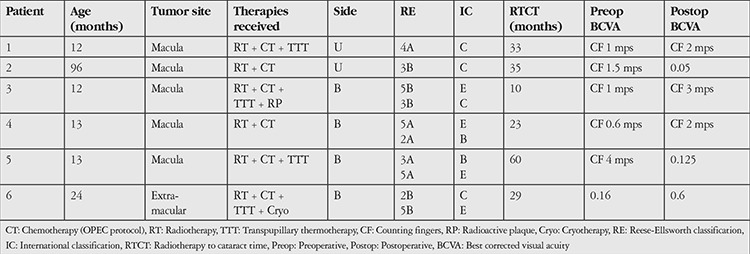
General patient characteristics
